# The Impact of Volunteer Wildlife Caregiving on Meaning in Life in Mid-Aged and Older Australians

**DOI:** 10.3390/bs16030381

**Published:** 2026-03-06

**Authors:** Claudia Scott, Nancy A. Pachana

**Affiliations:** School of Psychology, The University of Queensland, Brisbane, QLD 4306, Australia; claudiarscott@icloud.com

**Keywords:** meaning in life, wildlife caregiving, aging, volunteering, wellbeing, purpose, coherence, significance, Australia

## Abstract

Meaning in life is a key contributor to healthy aging, yet the mechanisms through which it is sustained remain underexplored. This study investigated the impact of volunteer wildlife caregiving on meaning in life among aging Australians, focusing on the three dimensions of coherence, purpose, and significance as conceptualized by the Three-Dimensional Meaning in Life framework. Eighteen participants (M age = 58.83, range 40–82; 13 female) completed a survey and semi-structured interviews about how their wildlife caring impacted coherence, purpose, and significance. Quantitative analyses revealed no significant differences in meaning in life scores by duration of volunteering. Qualitative thematic analysis, conducted using the Leximancer tool, indicated that coherence was supported by alignment with personal values such as past roles or conservation; purpose by passion, daily motivation, and identity; and significance by experiences of death and a sense of achievement. Findings highlight the centrality of animals in fostering meaningful, value-driven, and emotionally enriching experiences. Purpose and significance emerged as particularly salient for participants, reflecting motivational and evaluative aspects of meaning in life. These results demonstrate that wildlife caregiving can promote meaning in life, offering insights into strategies that may support such volunteering activities to contribute to aging well.

## 1. Introduction

Australia, like almost every other nation globally, is experiencing the aging of its population. Individuals aged 65 and over comprise more than 17% of the population (or approximately 5 million out of 27.6 million), a figure projected to rise to approximately 22% by 2066 (Australian Bureau of Statistics, 2025). This trend highlights the need to promote healthy aging and identify strategies to support Australians in leading fulfilling and meaningful lives as they age. Healthy aging is more than just the absence of disease; it involves developing and maintaining the functional ability that supports wellbeing in older age, encompassing both physical and mental health (World Health Organization (WHO), 2024).

A key contributor to aging well is the presence of meaning in life, which can be defined as reasons for living that motivate individuals in goal-directed activities (Feldman & Snyder, 2005). Previous research suggests that meaning in life has substantial relevance as a protective factor as people age, particularly in the face of life events such as retirement (Wood & Pachana, 2025) and evolving health and mental health concerns (Czekierda et al., 2017; Nie et al., 2023). Greater purpose and meaning in life have been associated with lower all-cause mortality rates and cardiovascular events (Cohen et al., 2016). However, although meaning in life is recognized as a critical factor in aging well, the mechanisms responsible for these relationships remain largely under-investigated (Hooker et al., 2018).

Despite the prominence of meaning in life as a concept, there is no singular consensus of its definition or an agreed-upon method of its measurement (see review by Heintzelman & King, 2014). Recently the construct has been viewed as multidimensional rather than unitary. Martela and Steger’s (2016) work identified three core pillars of meaning in life, namely viewing one’s life coherently, with purpose and significance.

According to Martela and Steger (2016), coherence is how one’s life makes sense as a meaningful and manageable whole, a consistency with values and a sense of self. It encompasses a connection to experiences and surroundings that reflect your identity and beliefs, and how one’s life fits together, with one’s past and present aligning. Purpose is about meaningful life direction and goals. This involves looking forward to the future and having overarching ambitions that provide meaning to daily actions, fostering a sense of fulfilment in life. Significance refers to the perception that life possesses inherent value and is worth living. It offers reassurance that one’s actions matter, fostering a sense of fulfillment and contribution rather than the feeling that efforts are futile. Building on this work, Martela (2017) highlights that significance is enhanced through engagement in activities that extend beyond the self. In this way, significance encompasses a sense of existential mattering, reflecting the belief that one’s existence holds value (George & Park, 2014).

Although coherence, purpose, and significance are distinct dimensions of meaning, coherence differs functionally from the other two components. According to G. Reker’s (2000) model, coherence is conceptualized as a cognitive component, whereas purpose and significance are regarded as motivational components. Coherence is descriptive and value-neutral, while purpose and significance are inherently evaluative, reflecting judgments about what is worthwhile (Heintzelman & King, 2014). Coherence represents the individual’s capacity to make sense of experiences and perceive the world as structured and predictable. In contrast, purpose and significance involve discerning what is valuable and imagining a future guided by meaningful goals. The pursuit of coherence entails constructing accurate mental representations that provide stability and consistency, whereas the pursuit of purpose and significance centers on justifying one’s actions, sustaining self-worth, and engaging in life paths deemed meaningful. Coherence is conceptualized as an epistemic notion, emphasizing knowledge and understanding, while purpose and significance are ethical notions, focusing on values and what is considered worthwhile (Martela & Steger, 2016). This distinction has been widely recognized within the meaning in life literature (Heintzelman & King, 2014; G. T. Reker & Wong, 2012; Steger, 2012).

Martela and Steger (2023) created the Three-Dimensional Meaning in Life Scale (3DM), which allowed for these components to be individually measured within one scale. This multidimensional framework was chosen as the theoretical foundation for the present study, as it offers a more comprehensive and operationalized understanding of meaning in life compared to earlier unidimensional models.

The human-animal bond provides an avenue to promote health and wellbeing across the lifespan (Pachana et al., 2011). Previous research has found that caring for pets can provide happiness, purpose and meaning in later life (Kalenkoski & Korankye, 2020) and that interacting with animals can positively affect health in older persons (Hughes et al., 2020). Emerging research suggests that fostering vulnerable companion animals may offer even greater benefits for adults as they age, as the reciprocal nature of the relationship and the sense of purpose it promotes beyond oneself are strong (Roseveare et al., 2023). Volunteering itself is well-established as a means to promote health and wellbeing in later life (e.g., Kim et al., 2020; J. W. K. Yeung et al., 2018).

Most research has examined the benefits of volunteering and caring for domestic animals. However, relatively little attention has been given to the effects of caring for animals outside the household pet context. Volunteer-based wildlife caregiving opportunities (where trained volunteers care for orphaned or injured wildlife) are widespread in Australia (Haering et al., 2018) and may offer similar or unique benefits.

One study that investigated the impacts of wildlife care was conducted by Englefield et al. (2019). Although this research did not examine effects on meaning in life, it highlighted how animal deaths can affect grief and overall wellbeing, with some caregivers experiencing burnout and compassion fatigue. P. Yeung et al. (2017) examined wildlife caregiving in New Zealand, reporting positive effects on quality of life. These findings suggest avenues for further investigation in an Australian context, particularly regarding meaning in life. A focus on wildlife care is especially relevant in Australia, given its unique biodiversity and frequent wildlife emergencies, such as bushfires and habitat loss. Across the country, numerous organizations provide opportunities for wildlife caregiving, largely sustained by volunteers, and may benefit from insights into the potential impacts on the wellbeing of volunteers in the second half of life.

This study focuses on caregivers who nurture a diverse range of species, including birds, reptiles, and marsupials, using Martela and Steger’s (2023) Three-Dimensional Meaning in Life Scale (3DM) to guide a qualitative exploration of the impact of volunteer wildlife care on meaning in life among mid-life and older Australians. Using respondents’ ranking of their three key components of meaning in life, namely coherence, purpose and significance, with respect to their wildlife caring responsibilities, this study shows which themes drive each dimension individually and indicate which is most important to caregivers. This research will be able to provide new insights into the specific mechanisms that drive meaning in life through volunteer wildlife care in aging adults.

## 2. Methods

### 2.1. Participants

Twenty-three registered wildlife caregivers were contacted to participate in the study, with nineteen subsequently completing the initial survey. One participant failed to complete the follow-up interview, resulting in a final tally of eighteen participants (M age = 58.83, age range 40–82, 13 females) completing the full study. Participants were roughly equally split in terms of level of education (high school, 8 participants, bachelor’s degree or higher, 10 participants) and current work status (9 participants each as retired/not working or in part-/full-time employment). The length of time spent volunteering for wildlife care was 14.82 (12.04), with a range of 0.67–39 years. Most caregivers (6 participants each) either did not specialize in any one sort of animal or cared for mammals exclusively; a smaller number cared for exclusively birds (4 participants) or reptiles (2 participants).

The inclusion criteria required participants to be aged 40 years or over, currently residing in Australia and currently actively involved (i.e., minimum 1 h per week) in volunteer wildlife caregiving. The age threshold was determined to accurately capture a broader portion of the wildlife caregiving community, as well as reflect a lifespan developmental perspective, since the purpose of the study was to explore meaning in the second half of life. All participants were registered with official wildlife caregiving associations (for example, Wildcare) and were recruited via networks arising from these organizations, including snowball sampling (Noy, 2008). The targeted sample was 20 based on the second author’s experience with similar studies, which successfully achieved data saturation, whereby interviewing further participants was unlikely to yield new insights. Ongoing analysis during data collection confirmed that saturation had been reached in this study; in other words, we deemed saturation reached as, despite the heterogeneity of caregivers, their reports of their experiences were remarkably similar, most likely due to the relatively niche volunteering experience of wildlife caregiving.

### 2.2. Measures

A study-specific online survey was developed to obtain demographic and relevant background information from participants, as well as to have participants complete the 3DM scale, which measures coherence, purpose and significance across eleven items. Responses are scored on a 7-point Likert Scale, ranging from *Not True at All* to *Very True*. An example item is: “I am highly committed to certain core goals in my life.” The 3DM scale allows for a tripartite view of meaning in life, and questions on the scale targeted each component separately. There are four items related to coherence, four related to purpose, and 3 related to significance. Final scores for coherence, purpose and significance were produced by calculating the average of the items corresponding to each domain for a participant. Within the brief format of this scale, it nevertheless demonstrates strong psychometric properties (e.g., α ≥ 0.90; Martela & Steger, 2023).

Following the online survey, participants were invited to join a brief online or telephone qualitative interview exploring these topics regarding meaning with respect to their wildlife caring in further depth. The interview initially began with the broad question “*Why is volunteer wildlife caregiving meaningful to you*?” to gain broader insights and establish rapport. This was followed by three separate queries: “*How does caring for animals influence coherence/purpose/significance in your life?*” as per each of the three components of meaning in life. Participants were provided with a succinct definition of each topic domain at the start of the interview to ensure that there was consistency in participants’ understandings of the components. After this, participants were finally asked to identify which component (coherence, purpose and significance) was most and least important to them, to gain a ranking of the three concepts, and to explain why they ranked them in this way.

### 2.3. Procedure

After gaining ethics approval from The University of Queensland Human Ethics Committee (2025/HE000558), potential participants were emailed a link to the initial survey, which included a Participant Information Sheet outlining the study purpose and procedures, and informed participants that completion of the survey would constitute implied consent. Upon survey completion the first author completed the audio-recorded interviews, which were conducted via Zoom or by telephone (67.7%), depending on participant preference. The interview format and order of questions remained consistent across both modes. All interviews were transcribed verbatim from the recordings. Each participant was assigned a unique identifier, and all data was de-identified to ensure confidentiality and anonymity and securely stored on the university’s Research Data Management System.

### 2.4. Data Analysis

Brief quantitative analyses on demographic data were undertaken to assess if there were differences in 3DM scores based on demographic factors such as gender and the length of time that participants had been volunteering with caring for wildlife.

**Figure 1:**
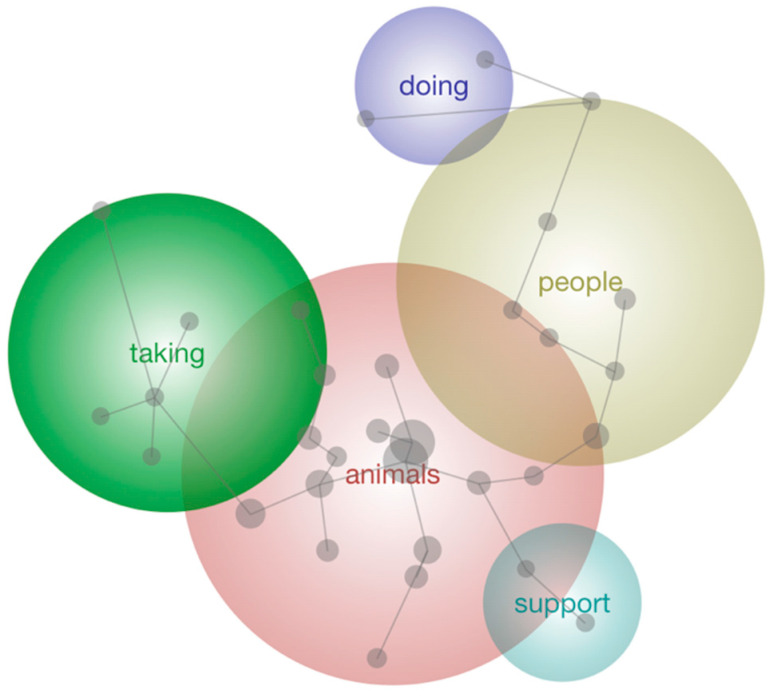
Themes discovered through Leximancer analysis relating to what is meaningful regarding volunteer caregiving. Note. Each theme label reflects the most salient concept within that cluster. The position, size and color of themes on the map indicate their relative importance in the dataset. Concepts that frequently appeared together in the data are displayed in closer proximity on the thematic map, with the degree of overlap between theme circles illustrating shared concepts. The map is heat-mapped on a gradient from warm to cool tones (red through yellow, green, blue and finally purple), where warmer colors highlight the most central themes. Connecting lines indicate relationships between concepts, and the size of the theme circles reflects the frequency with which they occurred in the data.

Qualitative data included transcribed responses describing the perceived importance of animal care as a whole, and then specifically of the three aspects of meaning in life (coherence, purpose and significance) analyzed in relation to various demographic factors and scores on the 3DM using Leximancer, a qualitative data thematic analysis software using machine learning techniques and nonlinear analysis (Leximancer version 5.0; Smith & Humphreys, 2006). Leximancer conducts thematic and semantic (relational) analyses of (in this case) interview data for the co-occurrence of words in the text to uncover meaningful patterns. These patterns are identified based on clusters of words that frequently appear together in the text. Guided by the researcher, it then uses a three-level network to build a layered semantic model composed of individual concept identification, clustering of themes and mapping of the relationships between themes. The output is presented as a concept map, in which the significance of a theme is indicated by the number of concepts it contains rather than the physical size of the theme bubble on the map. The relative importance of each theme is also conveyed through color: warmer tones indicate greater importance and cooler tones indicate lesser importance. Based on the qualitative data analyzed in this study, the theme size was adjusted until the clearest and most concise output was shown. A note of explanation on how to interpret the concept maps is provided in Figure 1.

## 3. Results

### 3.1. Quantitative Results

Objective 3DM scores indicated that significance (M = 5.98, SD = 1.28) was the most important dimension of meaning in life for the participants in this study, followed by purpose (M = 5.97, SD = 1.26) and then finally coherence (M = 5.69, SD = 1.25). While we did ask wildlife carers to rank the most and least important dimensions of meaning in life in the semi-structured interview, as several caregivers were equivocal in their responses, we did not analyze this data further.

Further analysis focused on whether differences in the length of time spent volunteer caring (range = 0.67–39 years) influenced scores on dimensions of meaning in life. Participants were separated into groups above and below the median length of volunteer time (Mdn = 11 years). Independent-samples *t*-tests found no significant differences between scores for those whose length of time volunteering was above compared to below the median in coherence, *t*(16) = −0.18, *p* = 0.85; purpose, *t*(16) = 0.11, *p* = 0.91; nor significance, *t*(16) = 0.44, *p* = 0.67. Similarly, scores did not differ significantly between men and women (coherence, *t*(16) = 0.75, *p* = 0.46; purpose, *t*(16) = 0.83, *p* = 0.42; significance, *t*(16) = 0.38, *p* = 0.71).

### 3.2. Leximancer Thematic Analysis

Qualitative output analysis of the initial survey question “What is meaningful to you about volunteer animal caregiving?” is shown in Figure 1. The central theme was *Animals* followed by *People*, *Taking*, *Support* and *Doing*. Theme size was set to 60%, which reflects the setting (between 0 and 100%) with respect to image granularity. *Animals* included the concepts Chance, Giving, Meaningful and Able. Participants described ideas within this theme, which related most strongly to animal care as, *“the most meaningful thing about caring for animals is saving their life,”*; *“you’re only giving them an hour or so of your life but that’s a lifetime to them.”*

*People* encompassed the concepts Work and Wildlife, with ideas within this theme relating most strongly to interpersonal interactions, with an example being, *“the group that I work with is a really lovely group, and so the interaction with them is really important.”* The next theme, *Taking*, incorporated the concepts Habitat, Area and Used. Participants described ideas within this theme, which related most strongly to loss of critical wildlife habitats, such as, for example, *“the poor animals are losing their habitat because we keep building houses and taking over their area.”*

The theme *Support* encompassed the concept Feeling and related most strongly to the relationship with the animal being cared for. An example of this was *“it’s a curiosity and learning. Getting the experience of understanding that animal.”* The final theme of *Doing* branched off the theme *People*, and included the concepts environment, wildlife and try. Participants described ideas within this theme, which related to making a change, as *“doing something to know that I contributed.”* Further analysis was conducted by separating responses according to the three pillars of meaning in life and analyzing them separately by topic.

**Figure 2:**
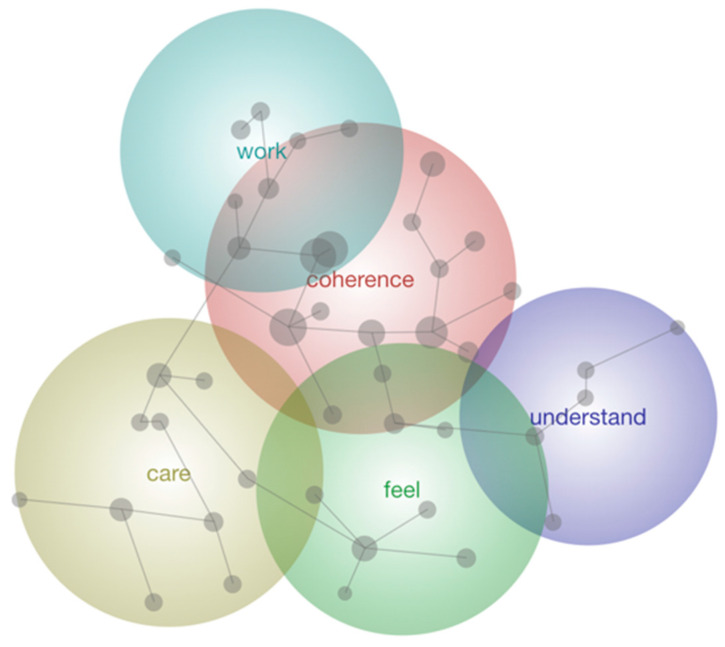
Leximancer-generated thematic map illustrating responses to “How does caring for animals influence coherence in your life?”.

Figure 2 presents the concept map of the most salient themes associated with coherence. The themes were rank ordered by Leximancer, with *Coherence* emerging as the most important, followed by *Care*, *Feel*, *Work* and *Understand*. Theme size was set to 49%.

*Coherence* was the central theme, from which all other themes branched, and it encompassed the concepts Animals, Caring, Life, Need and Role. Ideas within this theme, which related most strongly to coherence with respect to wildlife volunteering, were described by one participant as, *“Fulfilling where I lack in some other fundamental things in my life. Being able to [care for wildlife] fills a big void.”* Another participant explained: *“Caring for animals influences coherence as fits in perfectly with my life. It’s very coherent with my values.”*

The second theme, *Care*, involved the concepts Animals, Society and People. Ideas within this theme related most strongly to coherence with respect to caring more broadly. As one participant explained, *“I care for humans, and I care for wildlife, so I care for the environment. Learning and teaching others allow me to feel coherence.”*

The next theme, *Feel*, overlapped with care and incorporated the concepts Influences, Makes, and Difference, essentially relating back to ideas around making a difference. An example of this was, *“I feel very valuable, and I feel like I have added value to my life but also, to the planet.”* The theme of *Work* included the concepts World, Values, Important and Gives, relating back to ideas around working life, past and present. One participant described it as, *“Animal care drove me when I didn’t work anymore. Having wildlife has actually helped me come to terms with retirement.”*

The final theme within the topic of coherence was *Understand* and related to ideas around understanding/comprehension stemming from wildlife care; concepts here included Species, Conservation and Values. An example of this was, *“[I am] very enlightened to understand how incredible these beings are… You start getting the sense of the accomplishments and value in your life when you start looking at and learning about animals.”*

**Figure 3:**
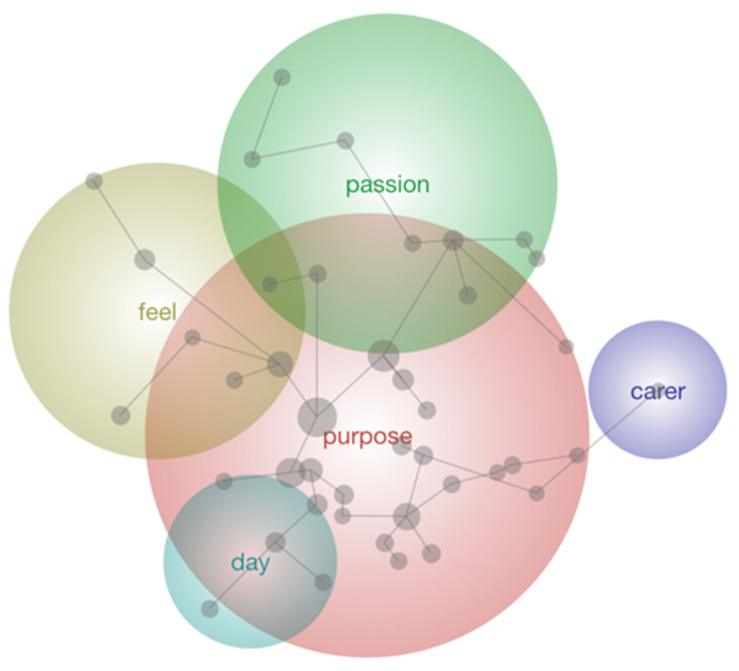
Leximancer-generated thematic map illustrating responses to “How does caring for animals influence purpose in your life?”.

Figure 3 presents the concept map of the most salient themes associated with purpose. When asked about the impact of their wildlife caring on purpose, the most important themes were *Purpose*, *Feel*, *Passion*, *Day*, and *Carer*. Theme size was set to 62%. *Purpose* emerged as the core theme, around which all other themes revolved and included the concepts Animals, Life, Doing and Able. Ideas within this theme related most strongly to purpose derived from wildlife caring. Some examples of this included *“I knew I had to find a purpose, a reason to get out of bed and earn my life”* and *“I don’t know how or what direction my life would be if I didn’t have the wildlife care in my life”.*

The next salient theme, *Feel*, encompassed the concepts Bird, Doing and Wild. Ideas within this theme related most strongly to purpose derived from giving back to nature or the wilderness. It was described by participants as: *“It has given me purpose as… I feel as though I can do anything that I put my mind to”* and *“Makes you feel like you’ve contributed. You’ve made a difference”.*

The next concept was *Passion,* which included the concepts Wanted, Goal and Time. Ideas within this theme related most strongly to purpose derived from their devotion and passion for wildlife care. One participant explained it as *“I’m very passionate and care about it… And I think I enjoyed being an engineer, but it wasn’t that level of passion.”*

*Day* followed and included Direction, Look and Things. Ideas within this theme related most strongly to purpose on a day-to-day basis, influenced by caregiving chores. An example here was offered by a participant, *“It’s given me purpose and something to direct my activities each day.”*

The final theme was *Carer*, and it was its own concept. Within this theme the ideas related most strongly to purpose are derived from the sense of identity derived from being a wildlife carer. An example quote from this theme was *“I have family, and I need to work but I think that I’ll always be a wildlife carer.”*

**Figure 4:**
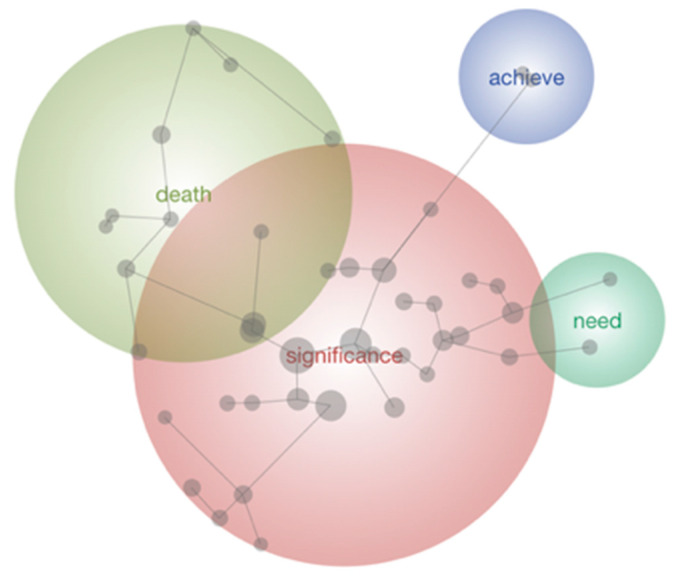
Leximancer-generated thematic map illustrating responses to “How does caring for animals influence significance in your life?”.

The concept map for the final core topic investigated, significance, is shown in Figure 4. The main theme was *Significance,* followed by *Death*, *Need* and *Achieve*. Theme size was set to 71%. The theme of *Significance* encompassed the concepts Animals, Life and Caring. Ideas within this theme related most strongly to significance derived from wildlife caring. Participants explained it as: “*You don’t only see them go, but they come back with their babies, and you know they’re succeeding and doing what they should be doing. That’s what it’s all about. It gives me significance to see them thrive.”* and *“There is a definite deeper kind of meaning to knowing that you are… directly helping wildlife survive.”*

The theme of *Death* followed, which included the concepts People, Die and Thought. It related most strongly to the relationship between animal care and death. *Death* was explained as “*so you should try to value your relationships, both with people and with animals, and pay more attention. Instead of hiding death off to the side, you have to face it.”* The next theme was *Need,* which involved the concept Everything; ideas within this theme related most strongly to the feeling of being needed each day. It was described as “Having a day-to-day routine and being responsible for animals gives a lot of significance. I do what I need to do because if I don’t look after my animals, it’s not going to be a good outcome.”

The final theme was *Achieve* and incorporated the concept Goals. Ideas within this theme are most strongly related to the sense of achievement wildlife care can provide. An example of this was *“you just keep getting the same goals of rehabilitating and releasing the animals. And that’s your short-term goal and you achieve that constantly.”*

While we did a comparison thematic analysis of men versus women (as there is a large body of literature on female caregiving experiences across people and animals) and retired versus working participants (as presumably their motivations might vary), neither of these comparisons revealed different thematic analyses. In other words, across men and women and working persons versus retired persons, the themes that emerged were comparable.

## 4. Discussion

The primary aim of the current study was to investigate the impact volunteer wildlife caregiving had on meaning in life among middle-aged and older Australians. It specifically focused on the factors that contributed to each of Martela and Steger’s (2016) three domains of meaning in life: coherence, purpose and significance. Qualitatively, the influence of the length of time engaged in wildlife caregiving on each domain of meaning in life was explored. Finally, qualitative interviews provided insights into how the experiences of wildlife caregivers related to their experience of meaning in life.

The quantitative analyses indicated that wildlife caregivers ranked significance as the most important dimension of meaning in life, followed by purpose and finally coherence. Length of time spent volunteering did not significantly affect 3DM scores. Previous research found that in older adulthood, short-term volunteering did not provide the same benefits to meaning in life as longer-term involvement (Jiang et al., 2020). Our findings suggest that even newer wildlife caregivers may experience meaning in life benefits. These findings were echoed by Milbourn et al. (2018), who suggested that there was no specific “ideal” length of time required for volunteering to be meaningful. Furthermore, research on human–animal interaction has shown that wellbeing benefits can occur after just a single encounter with an animal (Orr et al., 2023). This may explain the difference in findings from Jiang et al. (2020), as the present study focused specifically on animal-related volunteering.

In the qualitative analysis, participants described animals as central to the ways in which caregiving contributed meaning to their lives. Many reported that engaging in caregiving allowed them to positively influence the wellbeing of animals. Providing animals with a chance of survival and an opportunity to return to the wild was core. This is consistent with previous literature that denotes that a key characteristic and motivation of volunteer wildlife caregivers was their passion for animals (Kidd et al., 1996). Participants also emphasized the value of the social connections developed through caregiving, a finding consistent with other research highlighting the importance of social interaction for meaning in life (Krause, 2007). Wildlife caregiving provided them with a community they otherwise would not experience, which is especially important as social isolation becomes a greater risk with increasing age (Cotterell et al., 2018).

With respect to coherence, participants frequently described caregiving as consistent with their personal values. For example, one participant stated that caregiving *“fit in perfectly with my life,”* illustrating its integration with their sense of self. Some participants reported that wildlife caregiving allowed them to remain engaged in activities that preserved the values most central to former roles, such as teaching and nursing. This finding is consistent with work by Froidevaux and Hirschi (2015), which highlighted that sustaining meaningful roles after retirement is vital for maintaining a sense of meaning in life. For these participants, enacting actions reminiscent of their past careers helped the world “make sense” and reinforced coherence. Many participants emphasized that caring for wildlife allowed them to learn about different species and the consequences of extinction. This experience echoed findings by Vining (2003) that relationships with animals are a key motivator for environmental conservation.

Purpose was shaped by the importance of the development of passion, purposeful life engagement and the identity associated with being an animal carer. George and Park (2013) spoke of how purpose was an enthusiasm for the future, with passion representing a concrete expression of this enthusiasm. In the present study, wildlife caregiving was often described as igniting or deepening a passion, with many participants reporting that it was the only activity they felt a genuine commitment towards. Although they had engaged in other pursuits through employment, hobbies, or volunteering, participants emphasized that wildlife caregiving was uniquely sustainable because of the passion it fostered. Vallerand (2008) highlighted passion as essential to fostering purpose and to making life feel worth living.

Purposeful life engagement has positive effects on brain health and wellbeing in later life (Ryff et al., 2016). In this context, wildlife caregiving provided participants with structured, purposeful responsibilities that not only organized their daily lives but also contributed to a broader sense of life direction.

Finally, identity emerged as a notable contributor to purpose. Participants expressed confidence and authenticity in being recognized as a wildlife carer, describing the role as a reflection of their true selves. A loss of identity is just one of multiple losses people tend to experience when aging (Park, 2010). This finding resonates with research by Thoits (2012), who argued that a strong role-related identity enhances purpose and, in turn, meaning in life.

The final dimension, significance, was shaped by two central themes: death and the satisfaction of achievement. While achievement fostered a sense of contribution and impact, it was death that emerged as the most influential driver of significance. Participants emphasized that death is often overlooked in Western society, yet it is an unavoidable aspect of both animal care and life more broadly. Many reflected on how encounters with animal death deepened their appreciation for existing relationships and heightened awareness of life’s finitude. Experiences of death when caring for animals provided significance as it allowed caregivers to recognize the inherent value life provided. But death also takes its toll; both compassion fatigue and secondary post-traumatic stress disorder may affect wildlife caregiver mental health (Englefield et al., 2018).

Achievement also played a significant role in fostering significance. Participants described both the setting and attainment of goals as deeply important. The consistent experience of rehabilitating and releasing animals gave them a strong sense that their lives made a difference in the world. This aligns with Martela and Steger’s (2016) own work, which shows a strong correlation between accomplishment and meaning in life. The perceived importance of the task was irrelevant, even seemingly mundane achievements, such as in the case of this study, successfully feeding an animal, provided participants with the sense that their actions carried value beyond the immediate moment (Machell et al., 2014).

## 5. Strengths and Limitations

An important strength of the study is the use of the Three-Dimensional Meaning in Life Scale (3DM) by Martela and Steger (2023). This theory provided a strong framework to base the study around, offering three distinct pillars of meaning in life to be investigated. It was able to broadly capture the concept of meaning in life and apply it to volunteer wildlife carers. Measuring the factors individually using this scale allowed for nuanced insights rather than the vague findings that would be found when treating meaning in life as a singular multidimensional concept.

Limitations include that the sample of wildlife animal caregivers is a relatively small and homogenous sample and therefore may not be more broadly generalizable. As 13 of 18 participants in our sample were female, the findings may reflect a gendered perspective on wildlife caregiving. As mentioned by Kidd et al. (1996), the individuals who choose to volunteer as wildlife caregivers are likely to have similar morals, values and lifestyles. This homogeneity may limit the extent to which findings capture variability; for example, a very common theme throughout the research was the centrality of animals. As this study focused on a group so passionate about animals, other aspects that contribute to meaning may be overshadowed. The fact that our sample was confined to Australia means that findings cannot be generalized to other countries where conditions for volunteering in wildlife caregiving may vary. Cross-country comparisons represent a novel area for future research. Cultural, social, and national differences may influence how individuals derive meaning from this type of volunteering and which facets of meaning are most salient.

Further research with larger samples comparing wildlife caregivers with other general or specific volunteering groups may yield further interesting insights.

Finally, we note that the nature of our semi-structured interview questions appeared to pull comments about the more positive aspects of caregiving for injured or orphaned wildlife. Indeed, many participants highlighted how this work helped them with various aspects of their life—in terms of meaning, identity, feeling valued and so forth. This is not to suggest a romanticized view of wildlife caregiving; the risk of compassion fatigue and secondary post-traumatic stress disorder has been alluded to above (Englefield et al., 2018). But in this study, we asked, “*How does caring for animals influence coherence/purpose/significance in your life?*” and it appears from our albeit small sample that all aspects of that caregiving, even the death of the animal, have the potential to contribute to meaning in life.

## 6. Conclusions

By directly examining meaning in life among volunteer wildlife carers in the latter half of life, this study enhances understanding of what drives wildlife caregiving and how it supports meaning in life in aging Australians. Findings show that coherence is fostered through alignment with personal values and past roles, purpose through passion, daily motivation, and identity, and significance through experiences of death and achievement. Quantitative analyses indicate these benefits are consistent across gender, work status and volunteering duration. Overall, the results highlight the central role of wildlife caregiving in promoting meaningful, value-driven, and emotionally enriching experiences, with purpose and significance being particularly salient.

## Data Availability

Data available from corresponding author upon written request.
